# Whole-cell imaging of plasma membrane receptors by 3D lattice light-sheet *d*STORM

**DOI:** 10.1038/s41467-020-14731-0

**Published:** 2020-02-14

**Authors:** Felix Wäldchen, Jan Schlegel, Ralph Götz, Michael Luciano, Martin Schnermann, Sören Doose, Markus Sauer

**Affiliations:** 1 Department of Biotechnology and Biophysics, Biocenter, University of Würzburg, Am Hubland, 97074 Würzburg, Germany; 2 Chemical Biology Laboratory, Center for Cancer Research, National Cancer Institute, Frederick, MD 21702 USA

**Keywords:** Super-resolution microscopy, Membrane biophysics, Single-molecule biophysics

## Abstract

The molecular organization of receptors in the plasma membrane of cells is paramount for their functionality. We combined lattice light-sheet (LLS) microscopy with three-dimensional (3D) single-molecule localization microscopy (*d*STORM) and single-particle tracking to quantify the expression and distribution, and mobility of CD56 receptors on whole fixed and living cells, finding that CD56 accumulated at cell–cell interfaces. For comparison, we investigated two other receptors, CD2 and CD45, which showed different expression levels and distributions in the plasma membrane. Overall, 3D-LLS-*d*STORM enabled imaging and single-particle tracking of plasma membrane receptors with single-molecule sensitivity unperturbed by surface effects. Our results demonstrate that receptor distribution and mobility are largely unaffected by contact to the coverslip but the measured localization densities are in general lower at the basal plasma membrane due to partial limited accessibility for antibodies.

## Abstract (web summary)

The organisation of plasma membrane receptors is important for their function. Here the authors combine lattice light-sheet microscopy with 3D single-molecule localisation microscopy (*d*STORM) and single-particle tracking to study the distribution and motility of several surface receptors.

## Introduction

Since the beginning of the twentieth century, the role of receptors in modulating cellular processes has been studied extensively worldwide. Numerous drugs that specifically bind to receptors have been developed and are being used in established therapies^1^. To fully exploit the possibilities of receptor-targeted therapies, quantitative information about receptor expression, distribution, and mobility in the plasma membrane is required. Localization microscopy delivers single-molecule information about molecular compositions, spatial distributions, and about absolute numbers of proteins present in subcellular compartments^2^. Thus, important insights into the molecular organization of biological systems, e.g., the expression, distribution, and mobility of therapeutically addressable receptors in the plasma membrane of tumor cells, can be gained^3^.

Single-molecule imaging of plasma membrane molecules is commonly performed at the bottom plasma membrane at the coverslip/cell interface (referred to as the basal membrane) based on total internal reflection fluorescence (TIRF) microscopy^4,5^. However, such experiments have also raised questions to which extent the molecular organization and mobility of receptors on the basal plasma membrane is affected by contact to a glass surface. This has led to the use of light-sheet single-molecule microscopy to illuminate non-coverslip contacting regions and study membrane organization of T cells on the upper (apical) membrane^6,7^. Light-sheet illumination uses a separate set of optics to illuminate only a thin volume of the cell that corresponds to the focal plane of the detection objective, thus increasing the signal-to-noise ratio in single-molecule imaging and tracking experiments, and restricting photobleaching of dyes and photodamage of cells to a thin volume^6–8^.

The ideal light-sheet configuration enables whole-cell three-dimensional (3D) single-molecule localization and tracking, and excites only those molecules that can be detected at the same time. To fulfill these requirements, we used lattice light-sheet (LLS) microscopy^9–12^ in combination with single-molecule localization microscopy (SMLM), by direct stochastic optical reconstruction microscopy (*d*STORM)^13^ or photoactivated localization microscopy (PALM)^14^ and single-particle tracking for whole-cell 3D imaging of plasma membrane receptors^15,16^. LLS microscopy in combination with SMLM by points accumulation for imaging in nanoscale topography (PAINT) has already produced impressive images from thick samples including dividing cells and at the periphery of small embryos^10^. However, PAINT microscopy often leads to nonlinear swelling due to dye accumulation and requires acquisition times of up to several days, because labels continually bind to the specimen throughout the imaging process^10^. Although LLS-PAINT is suited for the study of densely crowded specimens, receptor imaging on the plasma membrane of whole cells does not exhibit such challenging conditions. Therefore, we use Alexa Fluor 647, the bridged carbocyanine dye Cy5B, and SeTau647-labeled primary antibodies for SMLM imaging and single-particle tracking, respectively, of plasma membrane receptors on fixed and living 293T cells. Although the carbocyanine dye Alexa Fluor 647 is the favorite dye for *d*STORM^17^, Cy5B fluorescence can be efficiently recovered from hydride reduction enabling PALM-like SMLM in oxygenated buffer with high localization precision^18^. The squaraine rotaxane dye SeTau647 exhibits a high photostability, enabling recording of long single-molecule trajectories^19^. The 3D whole-cell SMLM images of three different plasma membrane receptors (CD56, CD2, and CD45) show that contact to the coverslip does not induce receptor clustering. Receptor distribution and mobility are largely unaffected by contact to the coverslip. However, our data disclose that localization densities are in general lower at the basal plasma membrane, and CD56 and CD45 accumulate at cell–cell interfaces.

## Results and Discussion

### 3D-LLS-*d*STORM of plasma membrane molecules

**Fig. 1:**
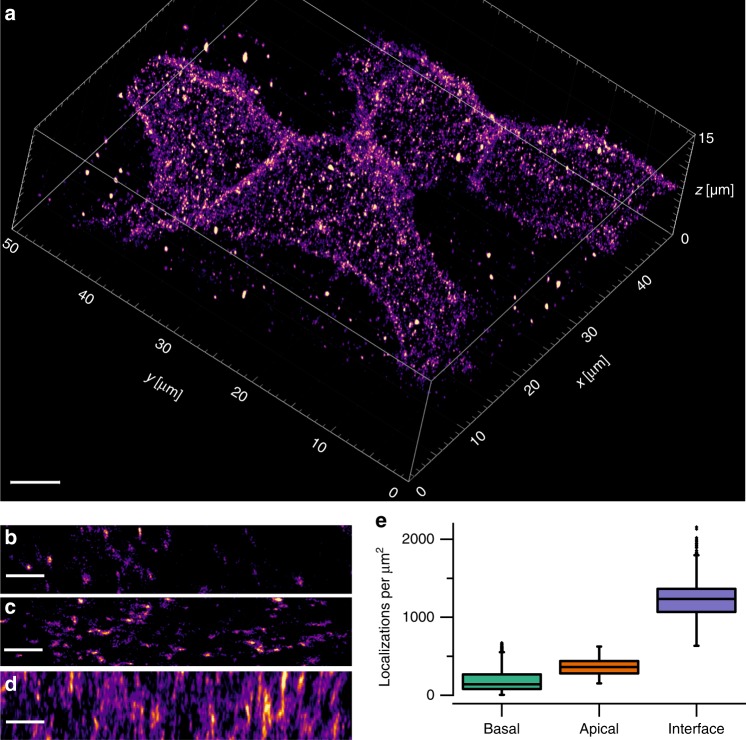
Visualizing the distribution of CD56 receptors in the plasma membrane of fixed whole 293T cells by 3D-LLS-*d*STORM. **a** Reconstructed volume rendering of single-molecule localizations shows considerable accumulation of CD56 receptors at cell–cell interfaces between the three cells. Representative 2D *xy*-projections of small areas of the basal (**b**) and apical (**c**) plasma membrane. Representative 2D projection perpendicular to the *z*-axis highlighting the increased CD56 density and the cell–cell interface (**d**). Note the lower spatial resolution in *z*-direction. **e** Localization densities (median ± MAD) measured at the basal (139 ± 76 localizations/µm^2^) and apical plasma membrane (361 ± 79 localizations/µm^2^), and at the cell–cell interface (1234 ± 146 localizations/µm^2^) for the shown cell. Boxplot of *n* = 7201 (basal), *n* = 854 (apical), and *n* = 628 (interface) sliding window data points. Boxplots show median (center line), 25th and 75th percentile (box), and 1.5× interquartile range (whiskers). Scale bars, 5 µm (**a**), 1 µm (**b**–**d**).

The neural cell adhesion molecule (NCAM), also known as CD56, is an important pathogen recognition receptor on human natural killer cells^20,21^ and is involved in fundamental biological processes including cell–cell adhesion, learning, and memory. To explore the distribution of CD56 on the plasma membrane of fixed cells unperturbed by coverslip interactions, we used astigmatic 3D-LLS-*d*STORM and Alexa Fluor 647-labeled primary antibodies (Supplementary Fig. 1). Using astigmatic 3D-*d*STORM in combination with cubic spline interpolated point-spread functions (PSFs)^16^, the axial detection range fully covers the axial irradiation volume defined by the light-sheet thickness of ~1.4 µm (Supplementary Fig. 2). To localize receptors on the whole plasma membrane of 293T cells, the sample was scanned through the LLS repetitively at 40 nm steps, while continuously acquiring images (Fig. 1, Supplementary Fig. 3, and Supplementary Movies 1 and 2). This resulted in a total acquisition time of 191 min for a volume of 53.1 µm × 26.6 µm × 40.0 µm (*x*, *y*, *z*), corresponding to a volume perpendicular to the coverslip of 47.5 µm × 52.5 µm × 11.7 µm in the “biological coordinate system” (*x*’, *y*’, *z*’) (Supplementary Fig. 1). We determined an experimental localization precision from more than 294,000 localizations of Alexa Fluor 647 blinking events of 16 nm in *x*, 17 nm in *y*, and 74 nm in *z*-direction, or when estimated after rotation to the biological coordinate system of 20 nm and 14 nm in the coverslip plane (*x*’, *y*’) and 38 nm in *z*’-direction (Supplementary Figs. 1, 4, and 5).

The 3D volume rendering of reconstructed localizations of 293T cells showed a heterogeneous distribution of CD56 receptors with a distinct accumulation at cell–cell interfaces (Fig. 1a and Supplementary Fig. 3a). Maximum-intensity projections of membranes illustrated the accumulation of CD56 receptors at cell–cell interfaces. The localization densities of CD56 at cell–cell interfaces of (1234 ± 146) and (2034 ± 350) localizations/µm^2^ (median ± median absolute deviation) was approximately twice as dense as it would be expected for two contacting apical membranes with densities of (361 ± 79) and (544 ± 80) localizations/µm^2^. The substantially lower localization densities measured at the basal plasma membranes of (138 ± 76) and (189 ± 97) localizations/µm^2^ indicate inefficient antibody labeling at basal cell membranes (Fig. 1e and Supplementary Fig. 3b).

For comparison, we measured the surface expression of two other plasma membrane receptors: CD2^22^, another cell adhesion molecule found on the surface of T cells, and CD45, the receptor-like transmembrane protein tyrosine phosphatase that is highly expressed on all nucleated hematopoietic cells^23^. The 3D-LLS-SMLM images showed that the expression of CD2 and CD45 on Jurkat T cells is lower than the expression of CD56 on 293T cells and is homogeneously distributed (Supplementary Figs. 6 and 7, and Supplementary Movies 3 and 4). A closer inspection of the localization densities revealed a higher CD2 density at the apical membrane (134 ± 25 localizations/µm^2^) compared with the densities at the basal membrane (23 ± 9 localizations/µm^2^) and a lower density at the cell–cell interface (76 ± 14 localizations/µm^2^) (Supplementary Fig. 6). As measured for the other plasma membrane molecules, CD45 showed localization densities of (47 ± 6 localizations/µm^2^) on the basal and slightly higher densities on the apical membrane (63 ± 8 localizations/µm^2^), but in contrast to CD2, a pronounced accumulation at cell–cell interfaces (218 ± 25 localizations/µm^2^) as also measured for CD56 (Supplementary Fig. 7). Overall, these data demonstrate that the measured localization densities are in general lower at the basal plasma membrane of adherent cells most probably due to inefficient labeling with IgG antibodies.

Strikingly, 3D-LLS-*d*STORM images and movies of CD56, CD2, and CD45 showed some smaller localization clusters that do not appear in 2D-*d*STORM images recorded from the basal plasma membrane by TIRF microscopy (compare Fig. 1 and Supplementary Figs. 3, 6, and 7). In 2D-TIRF-*d*STORM images, the receptors are homogeneously distributed and do not exhibit any sign of accumulation (Supplementary Fig. 8). Cross-sections of the basal, apical, and equatorial planes of CD2-, CD45-, and CD56-labeled cells and a close look at the 3D-LLS-*d*STORM movies showed that receptors are also localized intracellularly (Supplementary Fig. 9 and Supplementary Movies 1, 2, and 4), indicating that antibody binding induced endocytosis of receptors occurs (even though labeling was performed at 4 °C, see Online Methods) or antibodies might penetrate through membrane defects. The appearance of intracellular clusters in 3D-LLS-*d*STORM experiments depends strongly on the receptor investigated and typically varied between different experiments (Supplementary Fig. 9).

### Single-particle tracking by 3D-LLS microscopy

**Fig. 2:**
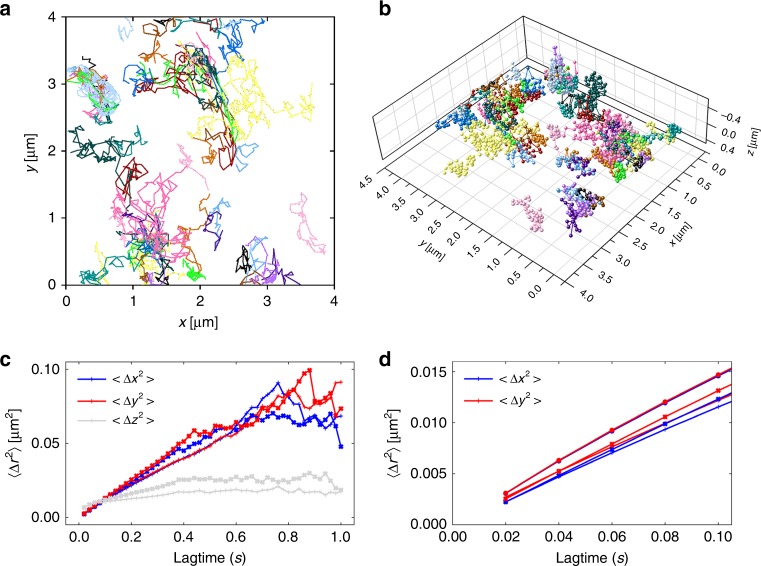
Single-particle tracking in living cells by LLSM. **a** Trajectories of CD56 molecules in the plasma membrane of 293T cells measured at 37 °C. **b** Astigmatic detection enables tracking of CD56 receptors in 3D. **c** The ensemble mean square displacement for each dimension *r*$\in$(*x*, *y*, *z*) for all trajectories with (cross symbols) and without (plus symbols) actin inhibitor CK666. *n* = 7 different cells were tracked for each condition. Each cell yielded an ensemble of >100 trajectories. **d** Ensemble mean square displacement zoomed to the part from which diffusion constants were determined by fitting a linear function. In addition to the LLS microscopy data (*n* = 7 tracking experiments examined from different cells of one biological sample), 2D-TIRF data are shown for 293T cells (*n* = 6 tracking experiments examined from different cells of one biological sample) adhered on cleaned coverslips (circle symbols).

Accumulation of receptors at cell–cell interfaces requires a high mobility in the plasma membrane. To investigate the mobility of CD56 in the basal plasma membrane of living 293T cells, we first performed two-dimensional (2D) single-particle tracking experiments using SeTau647^19^-labeled primary antibodies on poly-d-lysine-coated and untreated, cleaned coverslips by TIRF microscopy. In these experiments, the mobility of CD56 receptors showed a dramatic decrease when changing from cleaned coverslips to poly-d-lysine coating (Supplementary Fig. 10), which seriously complicates data analysis and interpretation. To exclude any interference of the measured mobility by surface effects, we used 3D-LLS microscopy to track CD56 receptors on the plasma membrane at 37 °C (Fig. 2). Single particles were tracked in 3D using astigmatic imaging, but without sample scanning. 3D-LLS microscopy allowed us to follow CD56 receptors on the fluid-exposed faces of the plasma membrane unperturbed by glass surface interactions. For quantification of diffusion dynamics, we analyzed the mean square displacement (MSD) < Δ*x*^2^ > in each dimension^15,24–26^. Although the overall data (Fig. 2) hints at some deviation from the linear time dependence that would be observed for free diffusion, we focused the quantitative analysis on the most reliable first five data points that could be fitted well to a model function for free diffusion. In living 293T cells, individual CD56 signals on the apical and equatorial plasma membranes exhibited a diffusion constant of 0.058, 0.059, and 0.023 µm^2^/s in *x*, *y*, and *z*, respectively, and offsets reflecting the localization precision of a few tens of nanometer (Fig. 2). Next, we added CK666 to the imaging buffer, a cell-permeable molecule that binds to the actin-related protein Arp2/3 complex and inhibits actin assembly^27,28^, and thus might influence the mobility of CD56 in the plasma membrane. However, we found only slightly increased diffusion constants of 0.06, 0.065, and 0.032 µm^2^/s, respectively, for the fast diffusion component. This effect is consistent with observations from fluorescence recovery after photobleaching (FRAP)^29^ experiments. FRAP data measured from cell–cell interfaces also indicated only minor insignificant variation in the measured time constants and the mobile fraction (Supplementary Fig. 11 and Supplementary Movie 5).

Overall, the NCAM CD56 works as a kind of glue that not only mediates cell–cell adhesion but also induces activation of a complex network of intracellular signaling cascades^21,22,30,31^. To fulfill its task, CD56 has to be mobile in the plasma membrane to move efficiently towards cell–cell contact areas and connect the two cells via trans-interactions. Using 3D-LLS microscopy in combination with SMLM (Fig. 1) and single-particle tracking (Fig. 2), we showed that CD56 receptors exhibit diffusional mobility in the plasma membrane and accumulate at cell–cell interfaces.

To summarize, we have demonstrated that 3D-LLS microscopy in combination with SMLM and single-particle tracking offers a unique and robust method to determine the distribution and mobility of plasma membrane receptors on whole cells unperturbed by surface effects. Tracking experiments in a LLS microscope will be helpful when studying cell types that do not adhere strongly on uncoated coverslides. Our results show that the labeling efficiency and mobility of plasma membrane molecules can be influenced by a direct contact to the coverslip (Figs. 1 and 2, and Supplementary Fig. 3) but their distribution in the membrane remains largely unaffected. Epi-fluorescence approaches have successfully demonstrated their potential for 3D SMLM deep within intact tissue using self-interference and active PSF shaping^32,33^; however, they irradiate the whole volume during the entire experiments and thus cause out-of-focus fluorescence background, reduced signals due to photobleaching of fluorophores, and photodamage in live-cell experiments. Hence, a combination of these methods with LLS microscopy will pave the way for quantitative, single-molecule sensitive 3D receptor imaging also within the tissue. Single-molecule sensitive 3D-LLSM approaches might be ideally suited to study the dynamic molecular organization of immunological synapses formed at the interface between antigen-presenting and T cells.

## Methods

### Cell culture and fluorescence labeling

The 293T cells (German Collection of Microorganisms and Cell Cultures, Braunschweig, Germany; #ACC635) were cultured in Dulbeccos’s modified Eagle’s medium (DMEM, Sigma-Aldrich, #D5796) with 10% fetal calf serum (FCS) (Sigma-Aldrich, #F7524), 1% l-Glutamine (already in DMEM suppl.), and 1% Pen-Strep (Sigma-Aldrich, #P4333) at 5% CO_2_ atmosphere and 37 °C. For single-molecule experiments, ~4 × 10^4^ cells were seeded onto poly-d-lysine-coated 5 mm coverslips the day before staining. The LEAF™ purified monoclonal mouse anti-human CD56 antibody (clone: HCD56, Biolegend, #318324) was conjugated with SeTau-647-NHS (SETA BioMedicals, #K9-4149) in 100 mM NaHCO_3_ buffer to obtain a degree-of-labeling (DOL) of ~1.7 and was stored in phosphate-buffered saline (PBS) with 0.05% sodium azide. Cells were washed with FluoroBrite™ DMEM (Gibco™ #A1896701) and labeled with 0.33 nM of the SeTau-647-conjugated antibody for 5 min at 37 °C. After two additional washing steps, single-particle tracking was performed in FluoroBrite DMEM supplemented with 170 µM CK666 (Sigma, #SML0006) or DMSO as control. For 3D-*d*STORM, cells were grown on poly-d-Lysine-coated 5 mm coverslips for 24 h. The CD56 antibody was conjugated with Alexa Fluor™ 647-NHS ester (Invitrogen™) in 100 mM NaHCO_3_ to obtain a DOL of ~2.3 as determined by absorption spectroscopy. Cells were washed and stained with 10 µg/ml of the Alexa Fluor™ 647-conjugated antibody in FluoroBrite on ice for 40 min. After two washing steps, cells were fixed with 2% formaldehyde and 0.2% glutaraldehyde for 15 min on ice and additional 15 min at room temperature followed by three additional washing steps. Jurkat T-cells were grown in RPMI 1640 media supplemented with 10% FCS, 1 mM l-glutamine, 100 U/ml penicillin, and 0.1 mg/ml streptomycin at 37 °C and 5% CO_2_. For imaging, 1.5 × 10^5^ cells per well were seeded in poly-d-lysine-coated chamber slides (Lab-Tek II, Nunc, Thermo Fisher Scientific) and allowed to adhere at 37 °C at 5% CO_2_ for 1 h. Fifty micrograms of monoclonal anti-human primary antibodies directed against CD2 (Biolegend; TS1/8, 309202) and CD45 (Biolegend, 2D1; 368502) were incubated in a 5 molar excess of Cy5B-NHS^18^ or Alexa Fluor™ 647-NHS (Invitrogen™), respectively, in 100 mM NaHCO_3_ at room temperature for 3 h in the dark. To remove unreacted dyes and to exchange the buffer to 0.02 NaN_3_ dissolved in PBS, the antibodies were purified with 0.5 ml 7 kDa Spin Desalting Columns (Thermo Fisher, 89882). Finally, the DOL of the purified antibody was determined by a UV-VIS spectrophotometer (Jasco V-650) to ~3–4. Conjugated antibodies were stored at 4 °C. Live-cell staining was performed on ice (~4 °C) for 45 min using an antibody concentration of 5 µg/ml in PBS. After washing, the cells were fixed for 15 min in 4% formaldehyde and 0.2% glutaraldehyde. Following three more washing steps, the cells were stored in PBS at 4 °C until imaging at room temperature.

### Lattice light-sheet microscopy

The LLS microscope was configured and operated similar to the one described in Chen et al.^9^ and Legant et al.^10^. To accommodate 3D-*d*STORM measurements and to benefit from commercial components, we made several modifications to the system. First, we used a 2 W 647 nm continuous wave laser (MPB Communications 2RU-VFL-p-2000-647-B1R) to produce an illumination intensity of 3.6 kW/cm^2^ at the sample for optimal photoswitching of Alexa Fluor 647^13,17^, and photoactivation and localization of Cy5B^18^. The laser was filtered by a clean-up filter (Chroma Technology Corporation ZET642/20×) and expanded to a 1/*e*^2^ diameter of 2.5 mm with a set of two lenses (Thorlabs C240TME-A and Edmund Optics 47-661). The spatial light modulator (Forth Dimension Displays QXGA-3DM) has smaller pixels (50.7 nm at the sample) and slightly faster switching times. For scanning of the light sheet, we used improved galvanometer mirrors (Cambridge Technology 8315K). The illumination objective (Special Optics, 0.70 numerical aperture (NA), 3.74 mm WD, part number 54-10-7) has a slightly increased NA. On the detection path, the system is equipped with a dichroic mirror (Chroma Technology Corporation ZT405/488/561/640rpc) and quad-band emission filter (Chroma Technology Corporation ZET405/488/561/640m). Single-molecule fluorescence signals were detected using a sCMOS camera (Hamamatsu Orca Flash 4.0 v3) connected to a water cooler (Innovatek AQ240-Pro), to eliminate vibrations. For single-color measurements conducted in this work, a long-pass filter (Chroma Technology Corporation ET655lp) was added directly in front of the camera. The pixel size at the sample was 103.8 nm. To further enhance the stability of the system, we built a custom light-tight enclosure around the whole microscope. Additional parts of the illumination and detection path were shielded with custom-made 3D printed parts to increase laser safety and light tightness. For illumination, a dithered, maximally symmetric fundamental square lattice with a minimum NA of 0.42 and a maximum NA of 0.50 was used, as it confines the excitation best to the depth of field of the detection objective (Nikon CFI Apo LWD 25XW, 1.1 NA), thereby reducing out-of-focus excitation and background fluorescence. At the same time, the light-sheet length of 15 µm, determined by the maximum NA, is ideally suited for the height of typical 293T cells. The light-sheet thickness was measured to 1.4 µm (Supplementary Fig. 2).

### 3D-*d*STORM imaging and density calculation

For *d*STORM imaging (Fig. 1), 293T cells were labeled as described above. The sample bath was filled with photoswitching buffer: PBS with 100 mM β-mercaptoethylamine (Sigma-Aldrich), 0.4% (w/v) glucose, and 0.4% glucose oxidase, adjusted to pH 7.4^13,17^. 3D-LLS-SMLM imaging of Cy5B-labeled receptors was performed in PBS buffer using solely 647 nm excitation^18^. Before imaging, the sample was reduced for 20–30 min in 26 mM NaBH_4_ and washed three times with 1× PBS. Upon irradiation at 647 nm, individual Cy5B molecules are spontaneously photoactivated and localized. Photoactivated fluorophores reside in their fluorescent state until they are photobleached^18^. Images were acquired at 50 Hz including the stepping motion, whereas the sample was scanned by 40 nm per frame in a saw tooth motion along *s* (*s* direction; Supplementary Fig. 1a). The stepping motion was performed during read-out of the camera. One thousand and one frames comprise one image stack, after which the acquisition repeats for a total of 573 stacks resulting in 1001 × 573 = 573,573 frames. SMLM processing was performed using the super-resolution microscopy analysis platform^16^ and the corresponding graphics processing unit (GPU)-accelerated Matlab software (available at https://github.com/jries/SMAP-light) after the measurement. Localization coordinates were corrected for sample scanning by subtracting the known shift. For 3D calibration, 100 nm fluorescent beads (TetraSpeck™ T7279, Thermo Fisher) were coated on a coverslip and imaged using the same buffer conditions. In Fig. 1, a total of 3.2 × 10^6^ localizations were detected of which 0.7 × 10^6^ were localized outside the 1.4 µm range illuminated by the light sheet or filtered with a minimum photon threshold and discarded. We did not use track emission to correct for overcounting. To correct for sample scanning during acquisition and to rotate the volume to coverslip plane, custom software was developed in Python. Rendering 2D images and histograms for density analysis was done using ThunderSTORM^34^ and FIJI^35^. To measure the densities at different parts of the cell membrane, a sliding window algorithm with a window size of 2 µm × 2 µm and a step size of 0.2 µm was implemented in FIJI. For rendering 3D volumes, Imaris (Bitplane) was used.

### Single-particle tracking

For single-particle tracking, cells were labeled as described above. The LLS microscope’s sample chamber was heated to 37 °C for at least 24 h before starting the experiments. For capturing single-particle dynamics, image sequences were acquired with 20 ms exposure time in a single plane. The data were fitted with a 3D PSF as already described and the resulting 3D molecule positions were linked to tracks using Trackpy^36^, a Python implementation of the popular Crocker–Grier algorithm^37^. For each measurement, the MSDs< Δ*x*^2^ >  of all tracks with a length of at least 20 frames were calculated and the resulting ensemble MSD was fit at small lag times with a linear function^15,24–26^< Δ*x*^2^ > (*τ*) *=* *o* *+* *2Dτ*

Here, *o* is an offset resembling the square of the localization precision, *D* is the diffusion constant, and *τ* is the lag time.

### FRAP analysis

The 293T cells were seeded into eight-well chambered cover glass (Cellvis, #C8-1.5H-N) and labeled after 24 h with 10 µg/ml Alexa Fluor™ 647-conjugated antibody in FluoroBrite for 10 min at 37 °C. FRAP experiments were performed on a confocal microscope (Zeiss LSM700) using the Plan-Apochromat 63 × 1.4 oil-immersion objective and 2% 639 nm solid-state laser excitation intensity for image acquisition. Every 1.5 s an image was recorded for a total acquisition time of 120 s and after three initial images a bleaching step was performed using 100% laser intensity of the 639 nm and 555 nm laser with a pixel dwell time of 37.6 µs. Frame size, bleaching area, as well as laser intensities and imaging speed was kept constant for all FRAP experiments. All experiments were performed at 37 °C and 5% CO_2_ using a stage top incubator (Tokai Hit). FRAP data were analyzed with the software Zen system 2012 and custom-made python code.

### Statistics and reproducibility

In all boxplots, the middle line is the median and the lower and upper hinges correspond to the first and third quartiles, respectively. The upper (and lower) whisker is drawn up to the largest (and smallest) observed data point within 1.5 times the interquartile range. Individual data points beyond the end of the whiskers represent all outliers. If not stated otherwise, 3D-LLS-*d*STORM data were acquired once from one biological sample, analyzed, and shown. Supplementary Fig. 10 depicts one from six and nine individual 2D single-particle tracking experiments examined from different cells of one biological sample for cleaned glass and poly-d-lysine coating, respectively. Supplementary Fig. 11 shows 1 from 12 individual FRAP experiments examined from different cells of one biological sample.

### Reporting summary

Further information on research design is available in the Nature Research Reporting Summary linked to this article.

## Supplementary information

Supplementary Information

Description of Additional Supplementary Files

Supplementary Movie 1

Supplementary Movie 2

Supplementary Movie 3

Supplementary Movie 4

Supplementary Movie 5

Reporting Summary

## Data Availability

All data that support the findings described in this study are available within the manuscript and the related Supplementary Information, and from the corresponding authors upon reasonable request.
