# Supplementary material for: Whole-cell imaging of plasma membrane receptors by 3D lattice light-sheet dSTORM
Source: Nat Commun. 2020 Feb 14;11:887. doi: 10.1038/s41467-020-14731-0 (PMC7021797; doi:10.1038/s41467-020-14731-0)
Supplement: Supplementary file 1 — Supplementary Information [file 41467_2020_14731_MOESM1_ESM.pdf]

Supplementary Information

Whole-cell imaging of plasma membrane receptors by 3D lattice light-sheet *d*STORM

Felix Wäldchen[1#], Jan Schlegel[1#], Ralph Götz[1], Michael Luciano[2], Martin Schnermann[2], Sören Doose[1] and Markus Sauer[1*]

[1]Department of Biotechnology and Biophysics, Biocenter, University of Würzburg, Am Hubland, 97074 Würzburg, Germany

[2]Chemical Biology Laboratory, Center for Cancer Research, National Cancer Institute, Frederick, MD 21702, USA

[#]These authors contributed equally: Felix Wäldchen, Jan Schlegel

Corresponding author: M.S. (m.sauer@uni-wuerzburg.de)

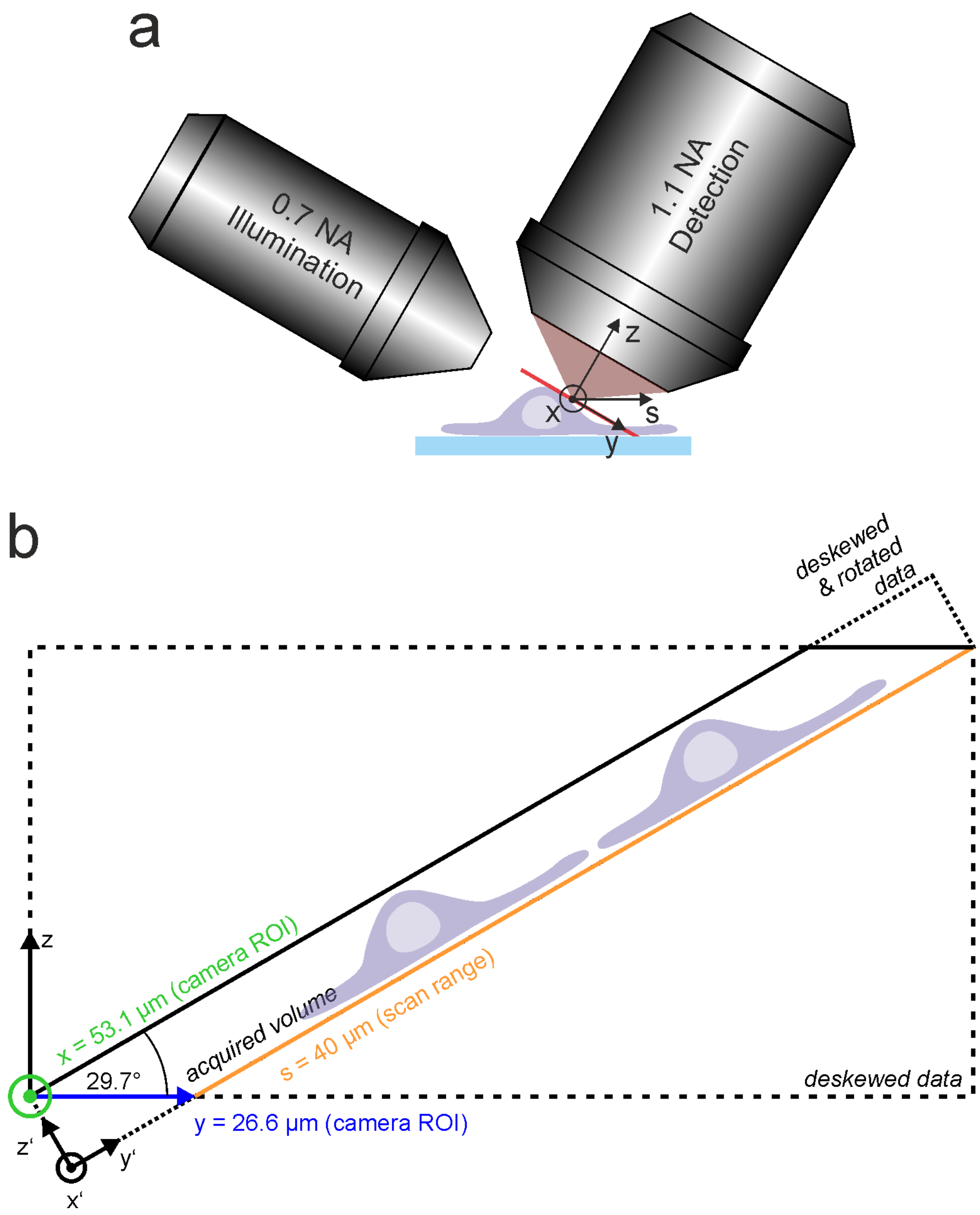

Supplementary Figure 1. Orientation and transformation of the lattice light-sheet coordinate system. (**a**) The illumination light propagates along the *y* axis and is spread out into a thin light-sheet (red) in *x* direction. Fluorescence light is collected perpendicular to the light-sheet (*z* axis). Cells (purple) that are adherent to the coverslip (blue) are scanned horizontally along the *s* axis during imaging, i.e. 3D *d*STORM measurements. (**b**) Deskewing the data is necessary to produce an image stack in rectangular coordinates. Rotating the deskewed data to the coverslip plane leads to a new coordinate system (x', y', z'). This biological coordinate system is used for the data of Figure 1.

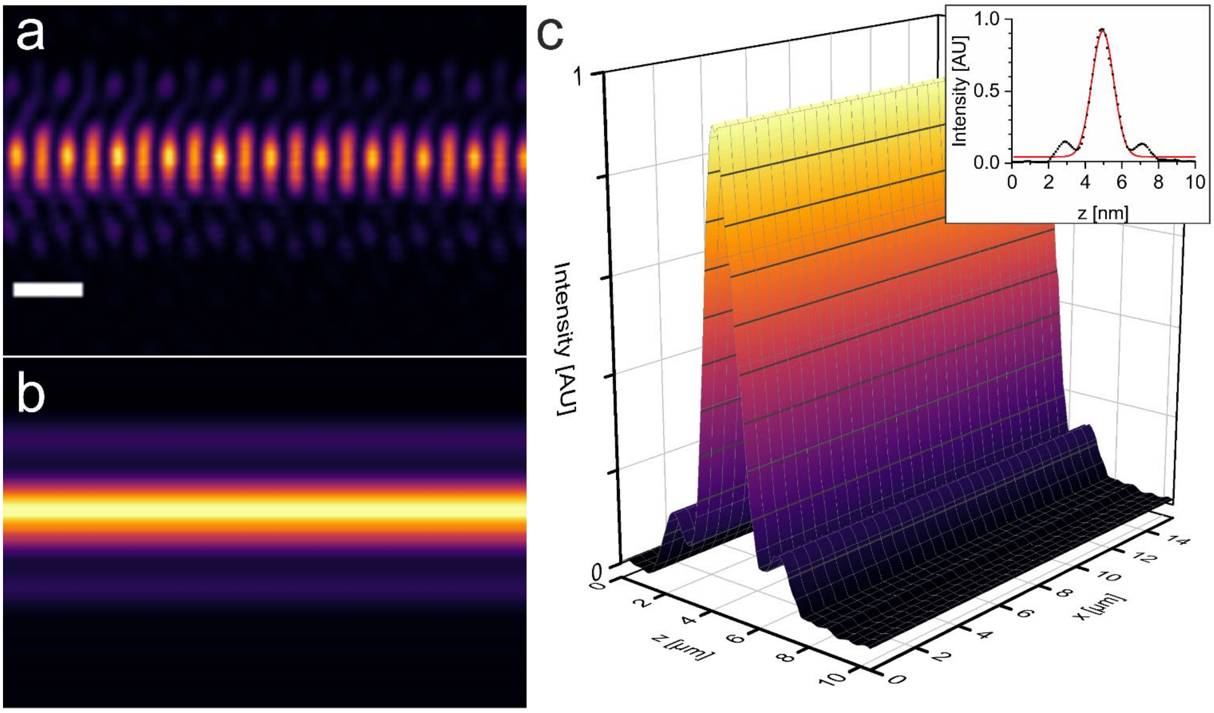

Supplementary Figure 2. Characteristics of the lattice light-sheet used in this work. (**a**) Experimentally acquired excitation PSF of the maximally symmetric fundamental square lattice in the *xz* plane. (**b**) Dithered excitation PSF calculated from (a). (**c**) Surface plot of the dithered excitation PSF to show the excitation efficiency. Fitting the cross-section with a Gauss function (inset) yields a light-sheet thickness of (1.40 ± 0.03) µm. Scale bar, 2 µm (a).

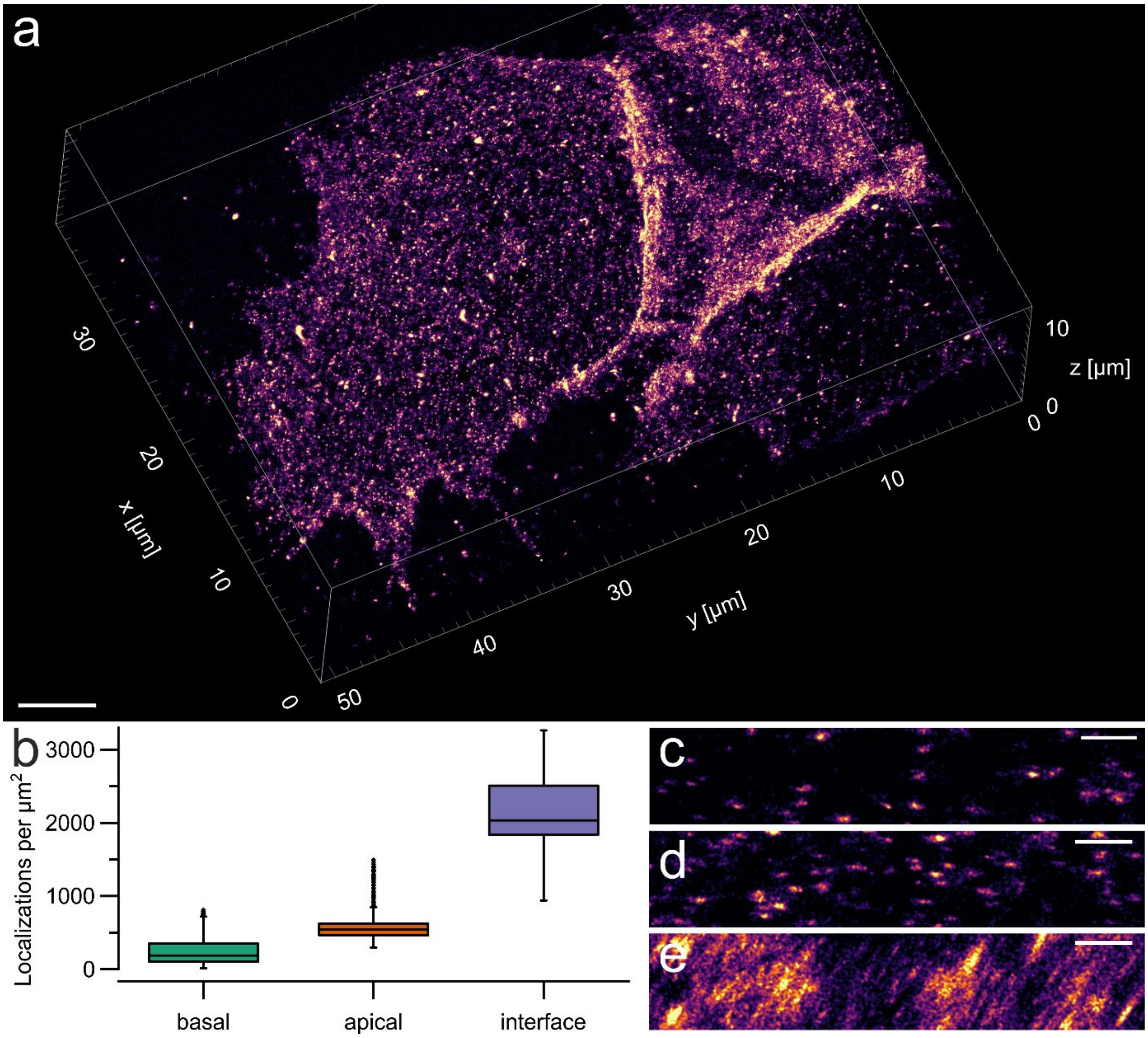

Supplementary Figure 3. Visualizing the distribution of CD56 receptors in the plasma membrane of fixed whole 293T cells by 3D-LLS-*d*STORM. (**a**) Reconstructed volume rendering of single molecule localizations shows considerable accumulation of CD56 receptors at cell-cell interfaces between the three cells. (**b**) Localization densities (median ± MAD) measured at the basal (189 ± 97 localizations/µm^{-2}) and apical plasma membrane (544 ± 80 localizations/µm^{2}), and at the cell-cell interface (2034 ± 350 localizations/µm^{2}) for the shown cell. Boxplot of n = 5610 (basal), n = 1188 (apical) and n = 270 (interface) sliding window datapoints. Boxplots show median (center line), 25th and 75th percentile (box) and 1.5x interquartile range (whiskers). Representative 2D *xy*-projections of small areas of the basal (**c**) and apical (**d**) plasma membrane. (**e**) Representative 2D projection perpendicular to the z-axis highlighting the increased CD56 density at cell-cell interfaces. Note the lower spatial resolution in z-direction. Scale bars, 5 µm (**a**), 1 µm (**b**-**d**).

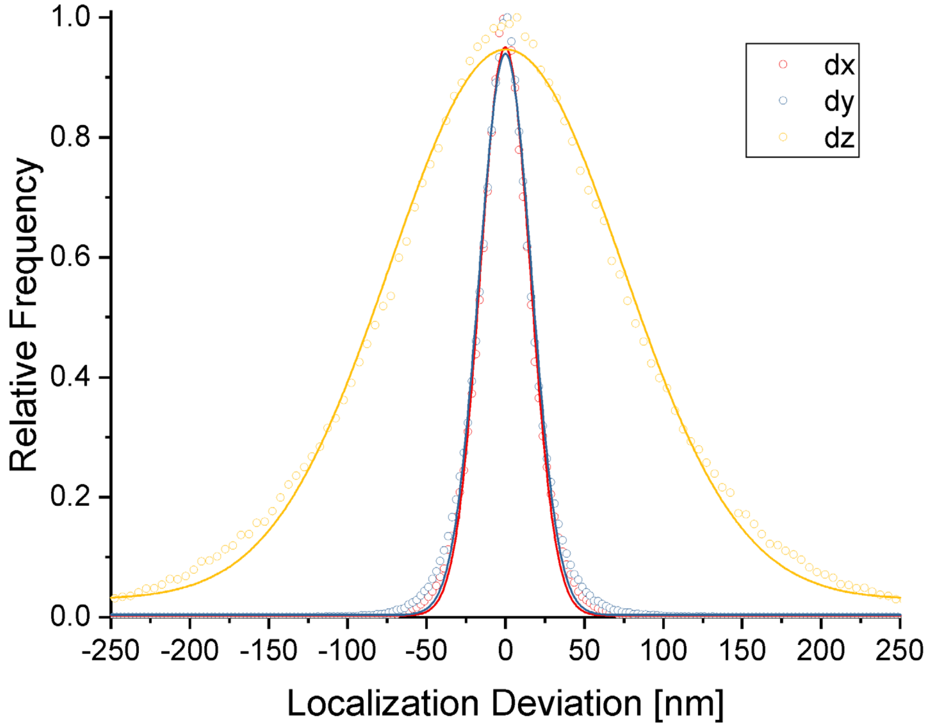

Supplementary Figure 4. Localization precision of 3D-LLS-*d*STORM with Alexa Fluor 647 of the data shown in Figure 1. n = 294,952 localizations were linked into tracks of at least 7 consecutive localizations using the Kalman filter implementation in *rapid*STORM[1]. The light-sheet intensity was homogeneous in the applied field of view. The deviation from the mean position of individual tracks was calculated and plotted as a histogram (data points). Fitting the histograms with Gaussian functions (solid lines) yields standard deviations of 16 nm, 17 nm, and 74 nm in *x* and y, and *z*, respectively.

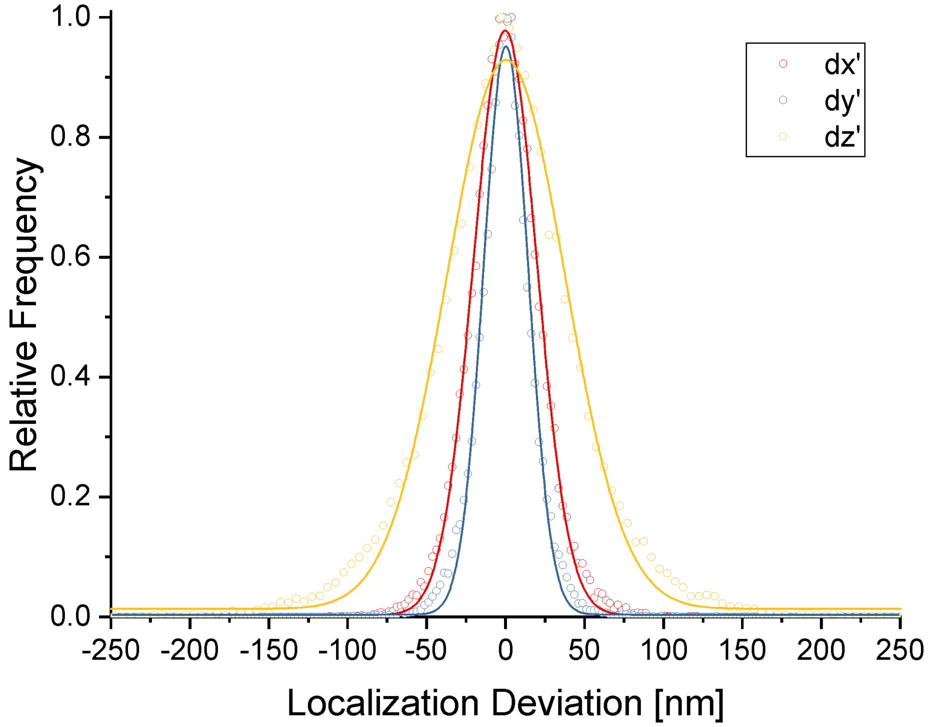

Supplementary Figure 5. Rotating the data set of Figure 1 to the coordinate system perpendicular to the cover slip (the biological coordinate system) merges localization precisions in x and z (Supplementary Fig. S1). The localization precision along x’ and y’ in the cover slip plane is 20 nm and 14 nm, respectively, while the precision along z’ perpendicular to the cover slip is 38 nm.

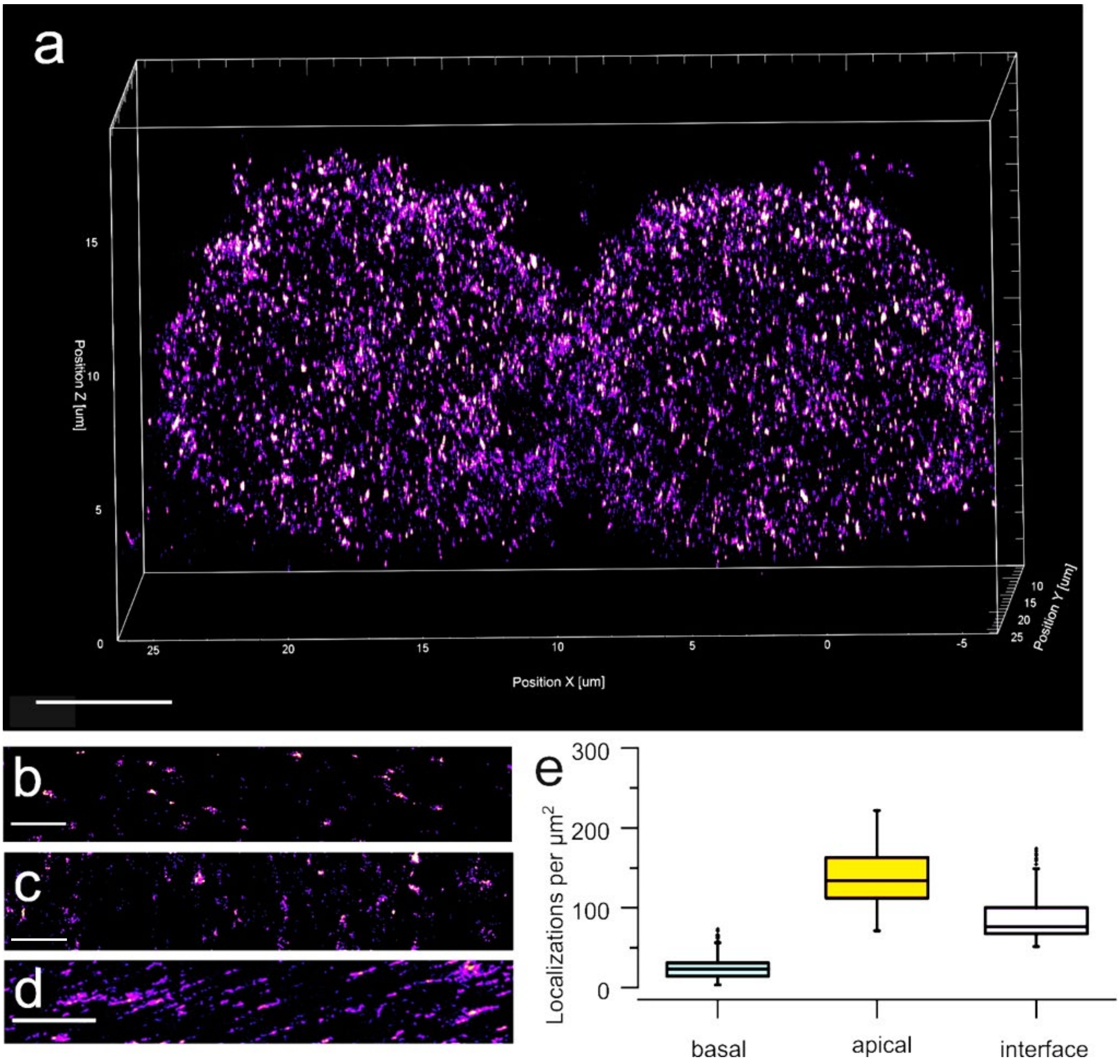

Supplementary Figure 6. Visualizing the distribution of CD2 in the plasma membrane of fixed whole Jurkat T cells by 3D-LLS-SMLM (single-molecule localization microscopy). Antibodies were labeled with Cy5B and SMLM was performed under reductive conditions[30]. (**a**) Reconstructed volume rendering of single molecule localizations shows a higher concentration of CD2 receptors at the apical membrane and a substantially lower concentration at the cell-cell interface between the two cells. Representative 2D *xy*-projections of small areas of the basal (**b**) and apical (**c**) plasma membrane, and (**d**) the cell-cell interface. (**e**) Localization densities (median ± MAD) measured at the basal (23 ± 9 localizations/μm^{-2}) and apical plasma membrane (134 ± 25 localizations/μm^2), and at the cell-cell interface (76 ± 14 localizations/μm^2) of the shown cell. Boxplot of n = 750 (basal), n = 434 (apical) and n = 144 (interface) sliding window data points. Boxplots show median (center line), 25th and 75th percentile (box) and 1.5x interquartile range (whiskers). Note the lower spatial resolution in z-direction. Scale bars, 5 µm (**a**), 1 µm (**b-d**).

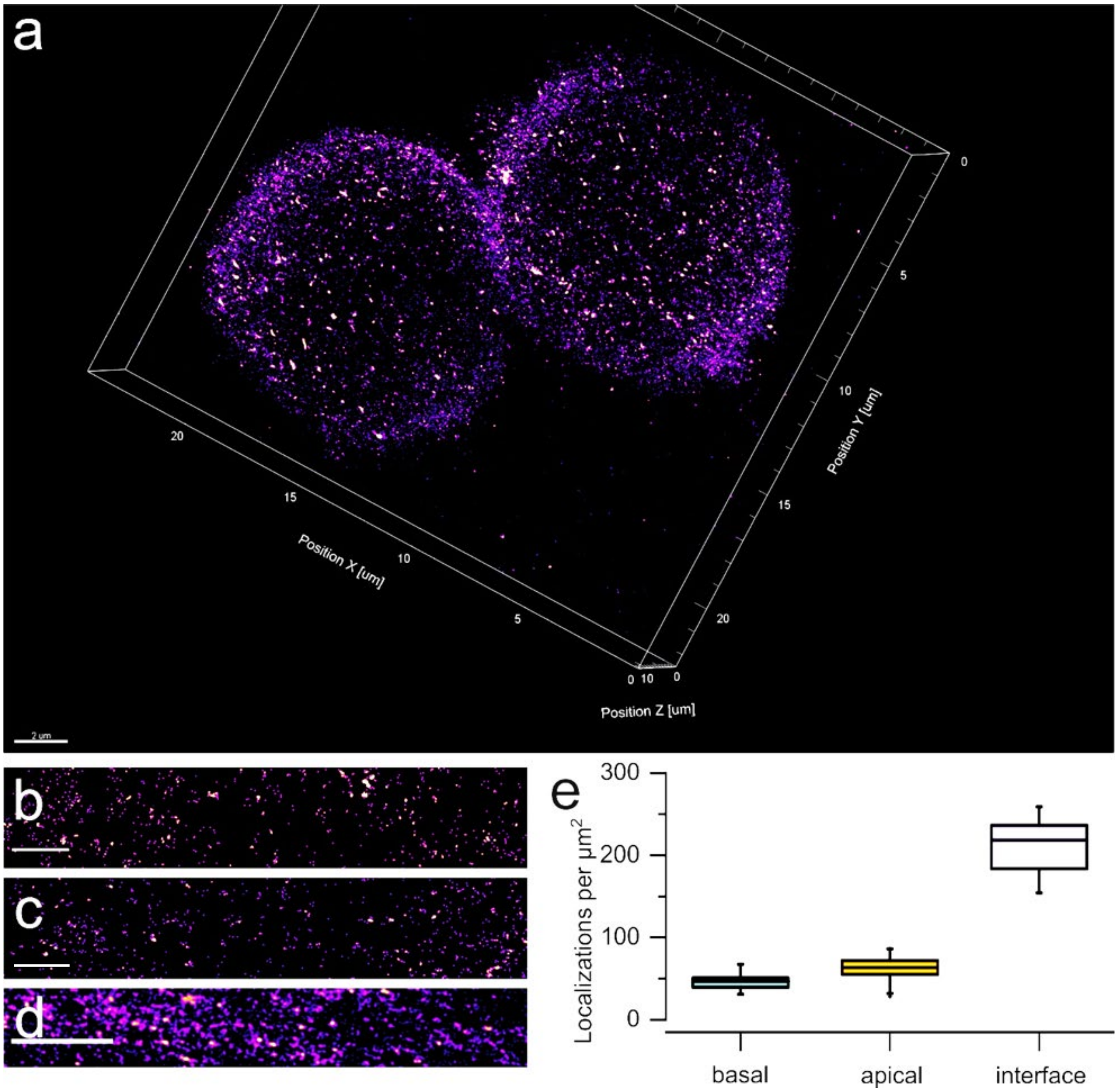

Supplementary Figure 7. Visualizing the distribution of Alexa Fluor 647 labeled CD45 in the plasma membrane of fixed whole Jurkat T cells by 3D-LLS-*d*STORM. (**a**) Reconstructed volume rendering of single molecule localizations shows a substantially higher concentration of CD45 receptors at the cell-cell interface between the two cells. Representative 2D *xy*-projections of small areas of the basal (**b**) and apical (**c**) plasma membrane, and (**d**) the cell-cell interface. (**e**) Localization densities (median ± MAD) measured at the basal (47 ± 6 localizations/μm^{-2}) and apical plasma membrane (63 ± 8 localizations/μm^{2}), and at the cell-cell interface (218 ± 25 localizations/μm^{2}) of the shown cell. Boxplot of n = 177 (basal), n = 322 (apical) and n = 80 (interface) sliding window data points. Boxplots show median (center line), 25th and 75th percentile (box) and 1.5x interquartile range (whiskers). Note the lower spatial resolution in z-direction. Scale bars, 5 μm (**a**), 1 μm (**b-d**).

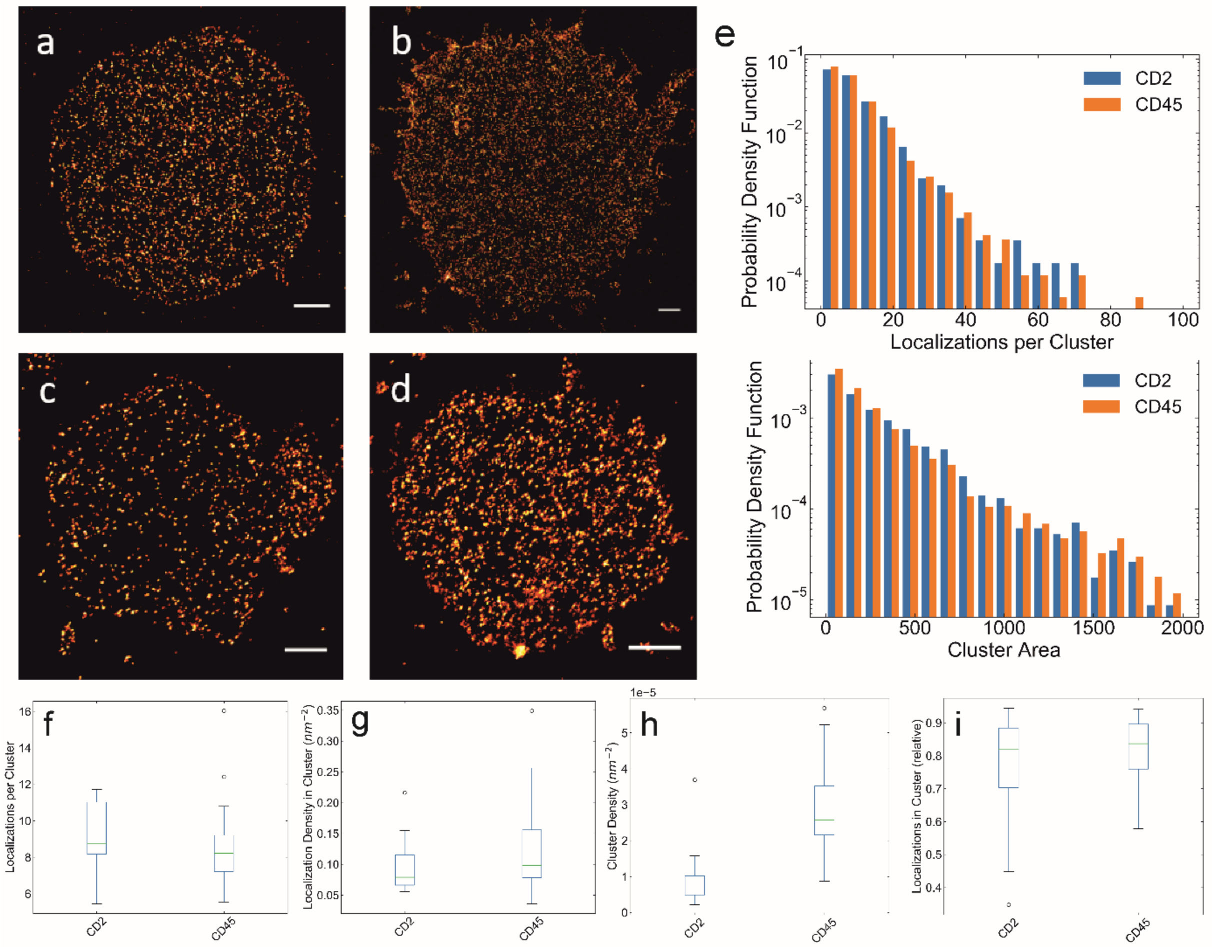

Supplementary Figure 8. 2D *d*STORM images of CD45 (**a**,**b**) and CD2 (**c**,**d**) recorded from the basal plasma membrane of Jurkat T cells by TIRF microscopy, respectively. Antibodies were labeled with Alexa Fluor 647. Both receptors are homogeneously distributed on the basal plasma membrane as confirmed by cluster analysis (**e**-**i**). (**e**) Probability density distributions of localizations per cluster and cluster area as determined from DBSCAN clustering for n=15 (CD2) and n=41 (CD45) cell regions. (**f**) The number of localizations per individual cluster. (**g**) The corresponding localization density in each cluster. (**h**) The overall cluster density per cell. (**i**) The relative number of all localizations that were included in cluster analysis. Scale bars, 1 µm.

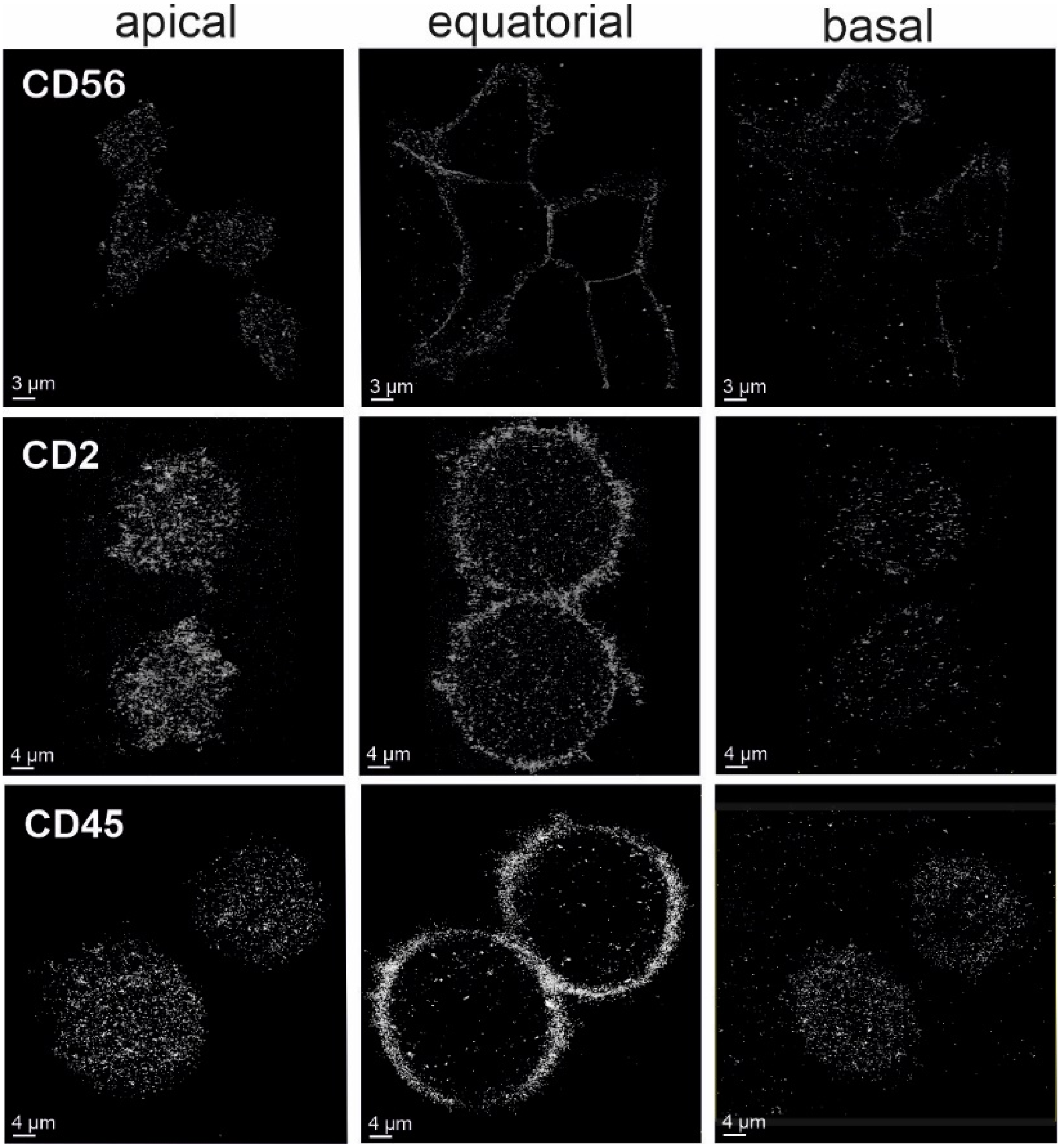

Supplementary Figure 9. Cross-sections of whole-cell 3D-LLS-SMLM of CD56, CD2, and CD45 on fixed Jurkat T and 293T cells, respectively, measured at the apical, equatorial, and basal plane. The cross-sections exhibit an axial thickness of ~1 µm. The images of the equatorial plane show also intracellular localization clusters most probably resulting from antibody binding induced internalization of receptors or penetration of antibodies through membrane defects.

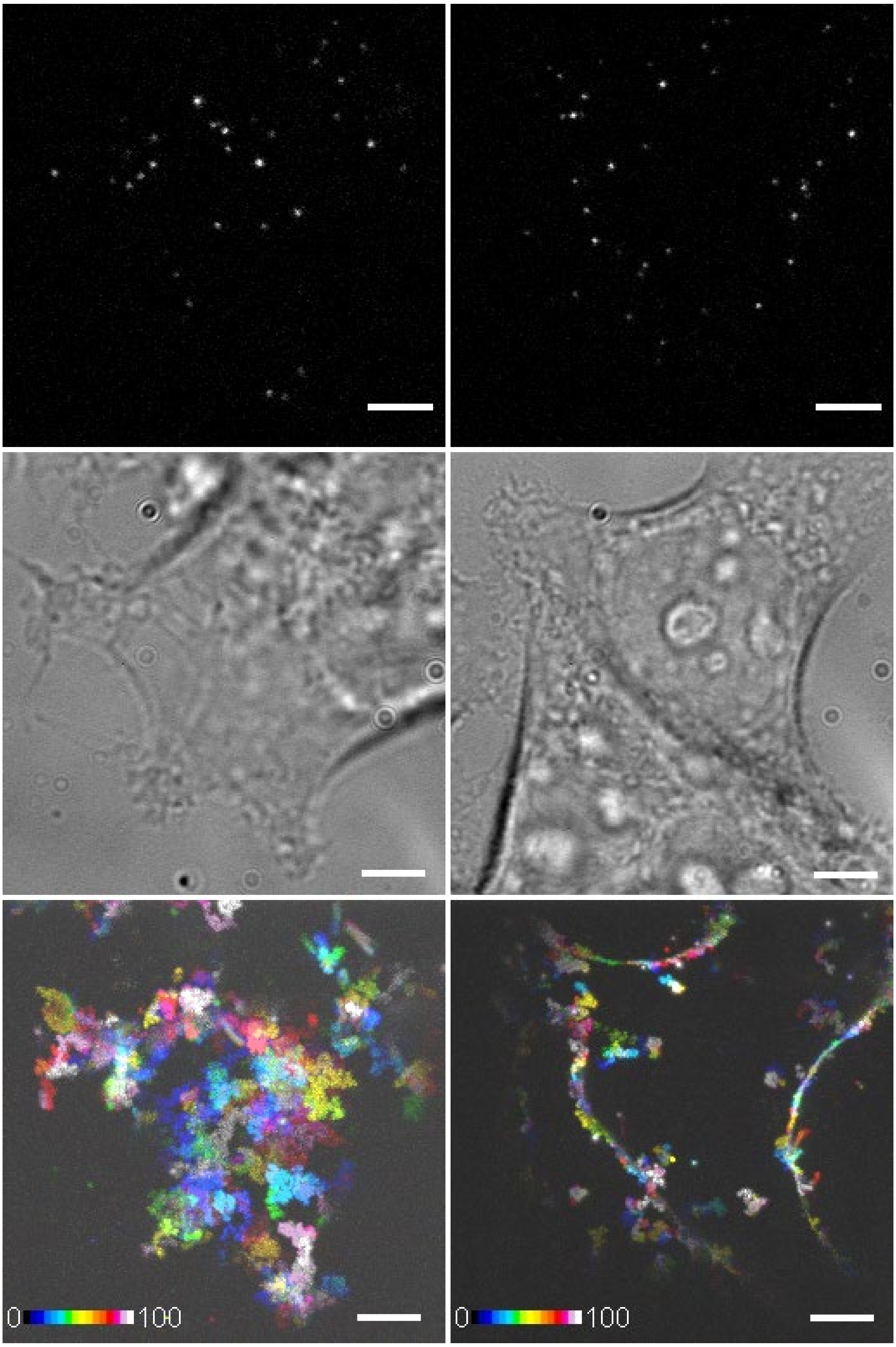

Supplementary Figure 10. 2D single particle tracking of CD56 at the basal membrane of 293T cells. Typical frames of single particle tracking videos (upper row), bright field images of cells investigated (middle row), and maximum intensity projections of unprocessed single-particle trajectories (color code ranging from 0 to 100 s) (lower row). Coating the coverslip surface with poly-D-lysine impedes fluorescence labeling with SeTau647-antibodies (right panel). Therefore, single-molecule tracks of CD56 can only almost exclusively recorded from perimeter membrane areas (right panel). Scale bars, 4 µm.

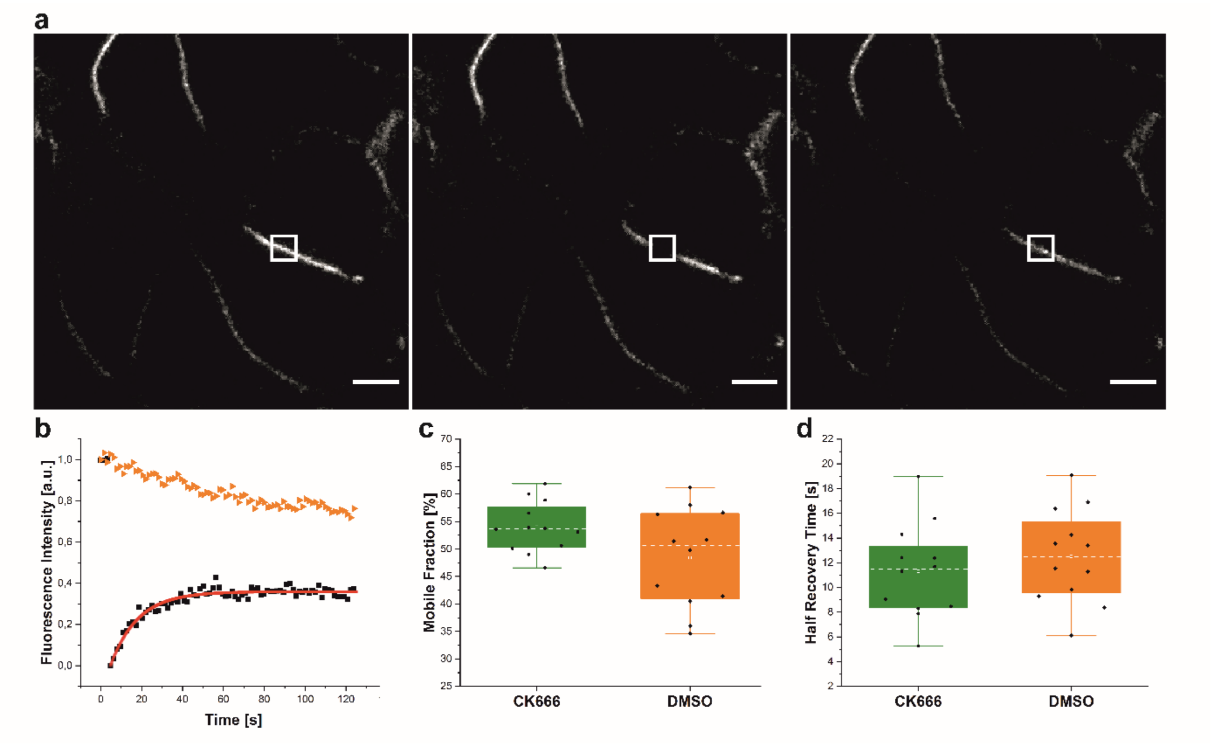

Supplementary Figure 11. FRAP experiments of CD56 at cell-cell interfaces. (**a**) Fluorescence images recorded before bleaching (left), immediately after bleaching (middle), and after recovery (right) of CK666 treated cells (see also Supplementary Video 5). Scale bars, 5 µm. (**b**) Representative FRAP curve from a single time series of DMSO treated control cells. Background- and bleaching-corrected data points are represented by black squares. Red line depicts the exponential fit. Orange triangles show the normalized signal over time from a non-bleached region. (**c**) CD56 was labeled with primary Alexa Fluor 647 conjugated antibodies and recovery analyzed in the presence of 170 µM small molecule inhibitor CK666 or DMSO as control. Median: 50.6% (DMSO) 53.7% (CK666) represented by dotted white line. White squares represent the mean of 12 FRAP experiments respectively. (**d**) CK666 treatment had no significant effect on the half recovery time of CD56 molecules. Median: 12.5% (DMSO) 11.5% (CK666) represented by dotted white line. White squares represent the mean of 12 FRAP experiments respectively. A two-sided t-test yields insignificant differences for (**c**) and (**d**).

Supplementary References

1. Wolter, S., et al. *Nat. Methods* **9**, 1040-1041 (2012).
