# Supplementary material for: Whole-cell imaging of plasma membrane receptors by 3D lattice light-sheet dSTORM
Source: Nat Commun. 2020 Feb 14;11:887. doi: 10.1038/s41467-020-14731-0 (PMC7021797; doi:10.1038/s41467-020-14731-0)
Supplement: Supplementary file 2 — Description of Additional Supplementary Files [file 41467_2020_14731_MOESM2_ESM.pdf]

Description of Additional Supplementary Files

File Name: Supplementary Movie 1
Description: Whole-cell 3D-LLS-dSTORM visualizing the distribution of Alexa Fluor 647 labeled CD56 on the plasma membrane of fixed 293T cells.

File Name: Supplementary Movie 2
Description: Whole-cell 3D-LLS-dSTORM visualizing the distribution of Alexa Fluor 647 labeled CD56 on the plasma membrane of fixed 293T cells.

File Name: Supplementary Movie 3
Description: Whole-cell 3D-LLS-SMLM visualizing the distribution of Cy5B labeled CD2 on the plasma membrane of fixed Jurkat T cells.

File Name: Supplementary Movie 4
Description: Whole-cell 3D-LLS-dSTORM visualizing the distribution of Alexa Fluor 647 labeled CD45 on the plasma membrane of fixed Jurkat T cells.

File Name: Supplementary Movie 5
Description: FRAP of a CK666 treated 293T cell. CD56 labeled with SeTau647-antibodies recorded from the cell-cell contact surface area (Supplementary Fig. 10).
