# Supplementary material for: Whole-cell imaging of plasma membrane receptors by 3D lattice light-sheet dSTORM
Source: Nat Commun. 2020 Feb 14;11:887. doi: 10.1038/s41467-020-14731-0 (PMC7021797; doi:10.1038/s41467-020-14731-0)
Supplement: Supplementary file 8 — Reporting Summary [file 41467_2020_14731_MOESM8_ESM.pdf]

nature research

Corresponding author(s): Markus Sauer
Last updated by author(s): Jan 27, 2020

Reporting Summary

Nature Research wishes to improve the reproducibility of the work that we publish. This form provides structure for consistency and transparency in reporting. For further information on Nature Research policies, see Authors & Referees and the Editorial Policy Checklist.

Statistics

For all statistical analyses, confirm that the following items are present in the figure legend, table legend, main text, or Methods section.

n/a	Confirmed	
☒	☐	The exact sample size (*n*) for each experimental group/condition, given as a discrete number and unit of measurement
☒	☐	A statement on whether measurements were taken from distinct samples or whether the same sample was measured repeatedly
☐	☒	The statistical test(s) used AND whether they are one- or two-sided<br>*Only common tests should be described solely by name; describe more complex techniques in the Methods section.*
☐	☒	A description of all covariates tested
☐	☒	A description of any assumptions or corrections, such as tests of normality and adjustment for multiple comparisons
☐	☒	A full description of the statistical parameters including central tendency (e.g. means) or other basic estimates (e.g. regression coefficient) AND variation (e.g. standard deviation) or associated estimates of uncertainty (e.g. confidence intervals)
☒	☐	For null hypothesis testing, the test statistic (e.g. *F*, *t*, *r*) with confidence intervals, effect sizes, degrees of freedom and *P* value noted<br>*Give P values as exact values whenever suitable.*
☒	☐	For Bayesian analysis, information on the choice of priors and Markov chain Monte Carlo settings
☒	☐	For hierarchical and complex designs, identification of the appropriate level for tests and full reporting of outcomes
☒	☐	Estimates of effect sizes (e.g. Cohen's *d*, Pearson's *r*), indicating how they were calculated

Our web collection on statistics for biologists contains articles on many of the points above.

Software and code

Policy information about availability of computer code

Data collection	Lattice Scope; Andor Solis; Zen System 2012
Data analysis	rapidSTORM 3.3; TrackPy; SMAP; Fiji; IMARIS; Zen system 2012; Pyhton 3.7

For manuscripts utilizing custom algorithms or software that are central to the research but not yet described in published literature, software must be made available to editors/reviewers. We strongly encourage code deposition in a community repository (e.g. GitHub). See the Nature Research guidelines for submitting code & software for further information.

Data

Policy information about availability of data

All manuscripts must include a data availability statement. This statement should provide the following information, where applicable:

- Accession codes, unique identifiers, or web links for publicly available datasets
- A list of figures that have associated raw data
- A description of any restrictions on data availability

The datasets generated during and/or analysed during the current study are available from the corresponding author on reasonable request.
In all boxplots the middle line is the median and the lower and upper hinges correspond to the first and third quartiles. The upper (and lower) whisker is drawn up to the largest (and smallest) observed data point within 1.5 times the inter-quartile range. Individual data points beyond the end of the whiskers represent all outliers. If not stated otherwise, 3D-LLS-dSTORM data were acquired once from one biological sample, analyzed and shown. Supplementary Figure 10 depicts one from 6 and 9 individual 2D single particle tracking experiments examined from different cells of one biological sample for cleaned glass and poly-D-lysine coating, respectively. Supplementary Figure 11 shows one from 12 individual FRAP experiments examined from different cells of one biological sample.

Field-specific reporting

Please select the one below that is the best fit for your research. If you are not sure, read the appropriate sections before making your selection.

☒ Life sciences ☐ Behavioural & social sciences ☐ Ecological, evolutionary & environmental sciences

For a reference copy of the document with all sections, see nature.com/documents/nr-reporting-summary-flat.pdf

Life sciences study design

All studies must disclose on these points even when the disclosure is negative.

Sample size	Sample sizes were determined based on the estimates from preliminary experiments and similar studies in the previous manuscripts so that reasonable statistical analyses could be conducted.
Data exclusions	No data was excluded.
Replication	Experimental findings reported in this manuscript were reliably reproduced in several experiments. The number of experiments performed are given in the figure legends.
Randomization	Randomization was not relevant to this study as 3D distribution and mobility of investigated receptors was not known in advance.
Blinding	Blinding was not relevant to this study as 3D distribution and mobility of investigated receptors was not known in advance.

Reporting for specific materials, systems and methods

We require information from authors about some types of materials, experimental systems and methods used in many studies. Here, indicate whether each material, system or method listed is relevant to your study. If you are not sure if a list item applies to your research, read the appropriate section before selecting a response.

Materials & experimental systems

n/a	Involved in the study	
☐	☒	Antibodies
☐	☒	Eukaryotic cell lines
☒	☐	Palaeontology
☒	☐	Animals and other organisms
☒	☐	Human research participants
☒	☐	Clinical data

Methods

n/a	Involved in the study	
☒	☐	ChIP-seq
☒	☐	Flow cytometry
☒	☐	MRI-based neuroimaging

Antibodies

Antibodies used	CD56 (clone HCD56, LEAF purified), CD2 (clone TS1/8, LEAF purified) and CD45 (clone 2D1, LEAF purified) all from Biolegend, London, UK
Validation	All antibodies are validated by the respective vendors.

Eukaryotic cell lines

Policy information about cell lines

Cell line source(s)	HEK293T (German Collection of Microorganisms and Cell Cultures, Braunschweig, Germany; #ACC635), Jurkat T cells (German Collection of Microorganisms and Cell Cultures, Braunschweig, Germany; #ACC282)
Authentication	None of the cell lines used in this study were authenticated in our lab, as they were directly purchased from the internationally credible vendors.
Mycoplasma contamination	In our laboratory, the contamination of mycoplasma was regularly examined by PCR, and found no contamination was detected while we conducted experiments concerning this work.
Commonly misidentified lines (See ICLAC register)	No commonly misidentified cell lines were used.
